# Use of pethidine for percutaneous liver biopsy – a randomised, placebo-controlled, double blind study

**DOI:** 10.1186/s12876-015-0264-8

**Published:** 2015-03-19

**Authors:** Antony Pan, Mohammed Alansari, Ralf Lubcke, Martin Schlup, Merrilee Williams, Margaret Fraser, Sarah Buckingham, Michael Schultz

**Affiliations:** 1 Gastroenterology Unit, Southern District Health Board, Dunedin, New Zealand; 2 Department of Medical and Surgical Sciences, Dunedin School of Medicine, University of Otago, Dunedin, New Zealand; 3 Department of Medicine, Redcliffe Hospital, Brisbane, Australia

## Abstract

**Background:**

Percutaneous liver biopsy (PLB) is the “gold standard” in the diagnosis of liver diseases. A pilot trial has shown pethidine may reduce anxiety and the need for post-procedural pain relief. The aim of this study was to investigate the role of pre-procedural pethidine.

**Methods:**

A double-blinded, randomized, placebo-controlled trial was conducted to assess the need for pethidine prior to PLB. 98 patients were randomly assigned to receive either 50 mg pethidine i.v. (n = 48), or an equal volume of 0.9% normal saline (n = 50). PLB was performed with ultrasound guidance after adequate local anaesthesia with xylocaine. Patients were asked to self-evaluate pain experienced using a visual analogue score (0–10) immediately and an hour after PLB. Patients were then followed up 24 hours after the procedure to assess their pain score, retrospective pain score and willingness to have a repeat procedure.

**Results:**

Pethidine administration resulted in significantly lower pain scores (0.60 ± 0.1 vs 1.2 ± 0.2, p = 0.006) and required less analgesia (0% vs 10%, p = 0.03) immediately after PLB in comparison to the placebo group. There was no significant difference in the pain score and analgesia requirement one hour after the procedure, the pain score at 24 hours after procedure and retrospective pain score. 94% of all patients of either group are willing to under go a repeat liver biopsy (NS).

**Conclusions:**

The administration of pethidine routinely prior to PLB reduces the immediate post procedural pain but has no lasting effect and does not influence the patients’ decision making process for future investigations.

**Trial registration:**

ACTRN12614001194651, 13 November 2014

## Background

Percutaneous Liver biopsy (PLB) remains an essential tool in the diagnosis and management of parenchymal liver disease. It is still considered as the “gold standard” in the diagnosis, assessment and staging of various diseases.

As an invasive procedure, PLB has been associated with several complications including post-procedural pain [1]. Liver biopsy can be performed under premedication with an opioid alone or in combination with benzodiazepines. Important issues for the use of sedation are patient’s cooperation and breath holding which is critical to the successful and safe completion of the procedure. Untoward movement during the procedure when the needle is in the hepatic parenchyma can lead to a tear of the liver and increases the risk of bleeding. There is variation between centers in techniques and guidelines for liver biopsy [2,3], in our center we routinely give pethidine as pre-medication. A pilot trial has shown that pethedine may reduce anxiety and the need for post-procedural pain relief [4]. The aim of this study was to investigate the role of pre-procedural pethidine for percutaneous liver biopsy.

## Methods

### Study design and population

A randomized, placebo-controlled, double blind study approved by the Lower South Regional Ethics Committee and undertaken at Dunedin Public Hospital (DPH), New Zealand. DPH is a tertiary teaching hospital with 350 inpatient beds covering a population of more than 190,000 people over 32,000 square kilometers of land in the lower South Island of New Zealand [5].

**Figure 1:**
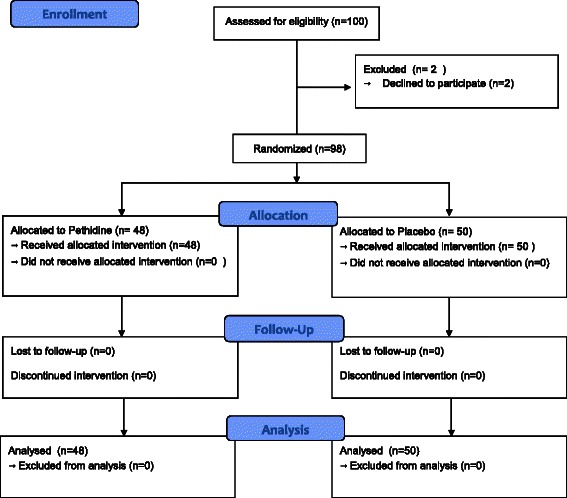
**Methodology flow chart.**

One hundred consecutive patients consented to be enrolled in the trial between March 2009 and May 2011, two patients withdrew their consent. All patients were given information sheet and written consents were obtained. Patients were randomly assigned to the pethidine (N = 48) or placebo group (N = 50) (Figure 1). The sample size was calculated to achieve 80% power to detect a 0.6 standard deviation difference in visual analogue score with an alpha of 0.05.

### Exclusion criteria

were as follows: 1) Patients with previous allergic reaction to pethidine or metoclopramide; 2) Patients with clotting disorders and abnormal prothrombin time with international normalized ratio (INR) of greater than 1.5 or thromcytopenia with platelet less than 50 × 10^9^ per litre; 3) abdominal ultrasound revealed a contraindication to PLB such as massive ascites, intrahepatic duct dilatation, or focal lesion; 4) patients without a telephone for study follow up; and 5) patients with a solitary lesion that required target biopsy under ultrasound guidance.

### Percutaneous liver biopsy

At Dunedin Public Hospital (DPH), a single consultant oversees the liver biopsies performed both by the consultant and advanced trainee in gastroenterology. Current blood results including full blood count and INR were reviewed and informed consent was obtained one day prior to the procedure. Patients were asked to fill in a Penn State worry questionnaire [6].

For the procedure, patients were placed in the supine position, and the ideal location for the liver biopsy on the lateral aspect of right thoracic site was established by both percussion as well as immediately prior to the investigation by abdominal ultrasound (4.0 MHz sector ultrasound probe; Acuson Aspen Advanced, Mountain View, CA 94043, USA). Patients were randomly (using randomization table) assigned to receive either 50 mg pethidine i.v. or an equal volume (1 ml) of 0.9% normal saline as premedication. All patients received 10 mg of metoclopramide i.v. The premedications were prepared by an independent nurse who was not involved with the care of patient before and after the procedure. The prepared medications were identical in volume and appearance and labeled with randomization number and patient details. The patients, assisting nurse and the operator were unaware of what premedication the patient received. The metoclopramide was given to mask the side effect of pethidine that patients may experience. Following disinfection of the skin with iodine, the patients were given the assigned premedication. The skin and subcutaneous layers of the thoracic wall were locally anaesthetized with 10 ml of 1% xylocaine containing 1:100,000 adrenaline. A small incision into the skin was made using a disposable scalpel. The liver biopsy was performed using a modified Menghini [7] biopsy set (17G X 70 mm Surecut, TSK Laboratory, Japan) using standard technique. Immediately following the biopsy the patients were placed on their right-side to rest on a sand bag for 1 hour, followed by an hour of lying flat. Blood pressure, pulse and wound site were checked regularly in the first hour. Lying and standing blood pressure were measured immediately prior to the procedure and two hours post procedure. All patients were observed for 4 hours after the procedure before discharge. Patients were asked to self-evaluate pain experienced using a visual analogue score (VAS, 0–10) at the end of procedure, and an hour after the procedure. Patients were then followed up with a telephone call 24 hours after the procedure to assess their pain scores, retrospective pain scores and willingness to have a repeat procedure if it was required in the future.

### Statistical analysis

The two groups were compared by Student t-test or Fisher’s exact test for qualitative variables. A *p* value of less than 0.05 was considered significant.

## Results

There were 63 male and 35 female patients with a mean age of 47.6 ± 8.5 years (range 17–81 years). The indications for PLB are hepatitis C (50%), hepatitis B (11.2%), autoimmune hepatitis (7.1%), alcoholic hepatitis (1%) and others (30.6%). Both groups were similar with respect to age, sex, body mass index and indication for liver biopsy. There were no significant differences in pre-biopsy anxiety score, current analgesic use, liver function test, INR, MELD (Model for End-stage Liver disease) score and number of passes to obtain a sample between the two groups (Table 1).

**Table 1:** **Clinical characteristics of the patients**

Characteristics	Pethidine group (n = 48)	Placebo group (n = 50)	P value
Age (SE)	46.9 (1.7)	48.3 (2.0)	NS (p = 0.60)
Sex ratio (M/F)	29/19	34/16	NS (p = 0.53)
BMI	27.7 (0.8)	28.2 (0.7)	NS (p = 0.68)
Indication			
Viral Hepatis C	31 (65%)	29 (58%)	NS (p = 0.54)
Other	17 (35%)	21 (42%)	NS (p = 0.54)
Anxiety Score	37.5 (1.5)	35.3 (1.7)	NS (p = 0.34)
Previous anxiety	8 (17%)	5 (10%)	NS (p = 0.34)
Previous biopsy (average)	0.14	0.27	NS (p = 0.19)
Current analgesic use			
Simple	7 (15%)	3 (6%)	NS (p = 0.20)
Opioids	6 (13%)	4 (8%)	NS (p = 0.52)
Laboratory			
INR (SE)	1.01 (0.01)	1 (0.02)	NS (p = 0.81)
Hemoglobin g/L(SE)	144.0 (2.1)	145.8 (10.6)	NS (p = 0.54)
Platelet × 10^9^/L(SE)	250.6 (11.6)	231.4 (10.6)	NS (p = 0.23)
Bilirubin micromol/L (SE)	10.5 (1.2)	11.3 (1.7)	NS (p = 0.73)
ALT u/L (SE)	85.8 (13.9)	72.6 (9.9)	NS (p = 0.45)
Creatinine micromol/L (SE)	78.8 (2.1)	82.6 (2.3)	NS (p = 0.23)
MELD score (SE)	7 (0.2)	7.2 (0.2)	NS (p = 0.47)
Procedure			
Number of passes	1.3 (0.07)	1.2 (0.06)	NS (p = 0.44)

Pethidine administration resulted in significantly lower pain scores immediately after the procedure than the placebo group: 0.60 ± 0.1 versus 1.2 ± 0.2 (p = 0.006). The range of pain score for the pethidine group was 0 to 2 compared to 0 to 4 in the placebo group.

No patient in the pethidine group required any additional analgesia immediately after the procedure in comparison to five (10%) patients in the placebo group (p = 0.03).

One patient from the pethidine group required pain relief compared to four patients in the placebo group an hour after the procedure however, there was no statistical difference in the pain score or pain relieve requirement an hour after the procedure.

There was no significant difference in the pain score and the retrospective pain score between the two groups at 24 hours after the procedure. 94% of the overall patients were willing to undergo a repeat PLB if clinically indicated; there was no significant difference between the two groups (94% vs 94%, NS) (Table 2).

**Table 2:** **Pain score (VAS) and analgesia requirement after PLB**

	Pethidine group (n = 48)	Placebo group (n = 50)	
**Immediately after procedure**			
VAS (SE)	0.60 (0.1)	1.20 (0.2)	p = 0.006
Analgesia requirement	0 (0%)	5 (10%)	p = 0.03
**1 Hour after procedure**			
VAS (SE)	0.23 (0.07)	0.46 (0.11)	NS (p = 0.08)
Analgesia requirement	1 (2%)	4 (8%)	NS (p = 0.19)
**24 Hour after procedure**			
VAS (SE)	0.88 (0.23)	0.63 (0.17)	NS (p = 0.40)
Retrospective VAS (SE)	2.20 (0.36)	1.5 (0.29)	NS (p = 0.13)
Willingness for repeat procedure	45 (94%)	47 (94%)	NS (p = 0.16)

There were five patients in the pethidine group and four patients in the placebo group with vasovagal reactions. There was no significant difference between the two groups in the side effects and complications. None of the patients developed severe complications such as bleeding, bile leak, pneumothorax or perforation of intraabdominal organs (Table 3).

**Table 3:** **Side effects and complications after PLB**

Complications	Pethidine group (n = 48)	Placebo group (n = 50)	
Nausea	0	0	NS (p = 1)
Vomiting	0	0	NS (p = 1)
Vasovagal reaction	5 (10%)	4 (8%)	NS (p = 0.74)
Serious complications	0	0	NS (p = 1)

## Discussion

Despite recent advances in non-invasive investigations, percutaneous liver biopsy (PLB) is still considered as the “gold standard” in the diagnosis, assessment and staging of various diseases. It provides an accurate diagnosis in approximately 90 percent of patients with unexplained liver function test abnormalities [8]. A significant degree of anxiety and high level of pain experienced by the patients [9] often limits the willingness of patients with chronic liver disease to undergo subsequent follow-up biopsies. Use of midazolam as sedative for frightened patients has been evaluated in the past [10,11], but its use in the clinical setting has been variable [2]. Sedation used in PLB may interfere with patients’ cooperation and possibly increases the complication rate. Recent published study shown combination of short-acting tramadol and lorazepam is effective, safe and can be used routinely before PLB [12]. Patient-administered nitrous oxide/oxygen inhalation has been evaluated as safe and excellent analgesia for PLB [13], but this is associated with high cost of the system, concern of personnel exposure to the gas, and the need for nursing supervision.

The attitude toward post-procedure pain and its prophylactic use of analgesics, anxiolytic, or sedative drugs is a matter of uncertainty and controversy. Consequently, there is variation between centers in techniques and guidelines for liver biopsy [2,3]. In our center we routinely give pethidine as pre-medication. A pilot trial from our center has shown that pethedine may reduce anxiety and the need for post-procedural pain relief [4]. The potential side effects of pethidine are considerable and include nausea, vomiting, sedation, dizziness, diaphoresis, urinary retention, and constipation. Overdose can cause muscle flaccidity, respiratory depression, hypotension, obtundedness and coma. The use of meperidine (pethidine) has been suggested as a safe analgesic option before percutaneous liver biopsy [14].

We conducted a randomized, placebo-controlled, double blind study to evaluate the need for pethidine in PBL. The results show that the use of pethidine provided a statistically significant pain relief in the immediate post procedural period in comparison to the placebo group but has not long-term effect.

In more detail, the mean VAS score was 0.6 in the pethidine group in comparison to 1.2 in the placebo group immediately after the procedure. Although there is statistically significant difference in the VAS score between the two groups, the VAS score of 1.2 in the placebo group is not considered as a clinically significant pain score as patients with VAS score of 2–3 are still usually quite functional.

Our results also showed that the use of pethedine had no long-term effect and did not impact on the pain score at one hour and 24 hours. With use of local anesthetic and good liver biopsy technique patients tolerated the PLB procedure with minimal pain.

More importantly, the result showed that overall 94% of patients were willing to undergo a repeat procedure if required regardless of whether they have received pethidine or not.

Administration of pethidine did not affect the rate of complications; there were no significant complications observed during the study period. These were consistent with our previous pilot study [11]. The use of pre-procedural ultrasound to identify the ideal location for all PLB, the usage of less traumatic Menghini technique and the supervision by a consultant may have contributed to the low complication rate [15]. There were four patients in the placebo group and five patients in the pethidine group who experienced vasovagal reaction with hypotension; all patients recovered spontaneously and were discharged within the same day of the procedure. The rate of vasovagal events was 10% in the pethidine group and 8% in the placebo group, this was higher than our previous reported rate of 1.3% [4].

## Conclusions

In conclusion, based on our results, we consider the use of pethidine as unnecessary and liver biopsy can be performed under good local anaesthesic technique. The use of pethidine before percutaneous liver biopsy resulted in better pain score and less analgesia requirement immediately after the procedure. However the use of pethedine had not impacted on the pain score and retrospective pain score at 24 hours after procedure. 94% of patients were willing to undergo a repeat procedure if required regardless of whether they have received pethidine or not. Pethidine use before procedure can be discussed with the patient; however its use may not enhance patients’ compliance with future biopsies.
